# Genome Sequence of *Gordonia* Bacteriophage Lucky10

**DOI:** 10.1128/genomeA.00580-16

**Published:** 2016-06-30

**Authors:** Welkin H. Pope, Aleks K. Brown, Daniel J. Fisher, Nicholas H. Okwiya, Kaitlyn A. Savage, Brian A. German, Jill E. McDonnell, Claire E. Schafer, Victor J. Yu, Emily C. Furbee, Sarah R. Grubb, Marcie H. Warner, Matthew T. Montgomery, Rebecca A. Garlena, Daniel A. Russell, Deborah Jacobs-Sera, Graham F. Hatfull

**Affiliations:** Department of Biological Sciences, University of Pittsburgh, Pittsburgh, Pennsylvania, USA

## Abstract

Lucky10 is a newly isolated phage of *Gordonia terrae* 3612 that was recovered from a soil sample in Pittsburgh, PA. Lucky10 has siphoviral morphology and a double-stranded DNA (dsDNA) genome of 42,979 bp, with 70 predicted protein-coding genes. Lucky10 shows little similarity to previously reported *Gordonia* phages.

## GENOME ANNOUNCEMENT

The bacteriophages of the phylum *Actinobacteria*, specifically those of *Mycobacterium smegmatis* mc^2^155, present a broad range of diversity at the genomic level (1). Relatively few phage genomes of *Gordonia* species hosts have been described, and their genomic diversity is poorly defined. *Gordonia* spp. have been implicated in the foaming of wastewater in treatment plants and as opportunistic pathogens in hospital infections (2–5). The Science Education Alliance-Phage Hunters Advancing Genomics and Evolutionary Science (SEA-PHAGES) program provides course-based undergraduate research experiences that can expand our insights into viral diversity and evolution using *Gordonia terrae* 3612 as a host for phage isolation and genomic characterization (3, 4, 6, 7).

*Gordonia* phage Lucky10 was isolated through enrichment of a soil sample obtained in Pittsburgh, PA. Electron microscopy shows that Lucky10 has siphoviral morphology, with an isometric head (50-nm diameter) and a tail 300 nm long. Following plaque purification and phage amplification, DNA was extracted and sequenced using Illumina MiSeq technology. Single-end 140-bp reads were assembled using Newbler into one major contig of 42,979 bp, with 65.4% G+C content and 396-fold coverage. The genome has discrete ends, with 3′-single-strand overhangs of the sequence 5′-CTCAGGGCAT. Seventy protein-coding genes were predicted using Glimmer (8), GeneMark (9), DNA Master (http://cobamide2.bio.pitt.edu), and Phamerator (10); functions were predicted for 23 of these by BLAST (11) and HHpred (12, 13) and alignment to the publically available databases GenBank, pFamA, and the Protein Data Bank. Lucky10 is not closely related to previously reported bacteriophages.

Most of the predicted Lucky10 genes are transcribed in the rightwards direction, with the exception of several genes, including a putative immunity repressor, near the center of the genome. The Lucky10 left arm contains the virion structure and assembly genes, including those encoding a capsid maturation protease (*4*), terminase large subunit (*2*), portal (*3*), major capsid subunit (*6*), major tail subunit (*12*), tape measure protein (gp16), and minor tail proteins (*17–19*, *25–27*). Several of the structural genes are related to those in cluster G mycobacteriophages, including the terminase large subunit, portal, and capsid subunit genes. The Lucky10 major capsid subunit has 83% amino acid identity to that encoded by cluster G phage MOOREtheMARYer (14), and the two also have 79% nucleotide sequence identity spanning 800 bp of the major capsid subunit genes. The lysis cassette is unusually situated among the minor tail protein genes.

Lucky10 encodes a putative tyrosine integrase (*35*) near the center of the genome, and we identified a potential *attP* common core at coordinates 27495 to 27521 within the intergenic region between positions *32* and *33*. This 26-bp sequence is shared by the putative *attB* site that overlaps a *Gordonia* tRNA^Ala^ gene (KTR9_RS18675 in *Gordonia* strain KTR9 [15]).

The Lucky10 right arm is replete with genes of unknown function, although functions could be assigned to a DNA methylase, a RusA-like Holliday junction resolvase, two DNA-binding regulatory protein genes, an exonuclease, and two homing endonucleases.

### Nucleotide sequence accession number.

The Lucky10 genome is available from GenBank under the accession no. KU963256.
